# Alpine songbirds at higher elevations are only raised with a slight delay and therefore under harsher environmental conditions

**DOI:** 10.1002/ece3.70049

**Published:** 2024-07-25

**Authors:** Julia Paterno, Fränzi Korner‐Nievergelt, Stefanie Gubler, Pia Anderwald, Valentin Amrhein

**Affiliations:** ^1^ Department of Environmental Sciences University of Basel Basel Switzerland; ^2^ Department of Research and Monitoring Swiss National Park Zernez Switzerland; ^3^ Swiss Ornithological Institute Sempach Switzerland; ^4^ Swiss Academy of Sciences SCNAT Bern Switzerland

**Keywords:** Atlas code, breeding stage, detectability, passerine, plant phenology, willow tit

## Abstract

The breeding phenology of birds is often timed to coincide with a peak in food availability. However, the shortening of the vegetation period with increasing elevation may force bird species at high elevations to breed earlier in relation to optimal environmental conditions due to time constraints. We investigated differences in fledging dates in five Alpine woodland songbird species along an elevational gradient from 1500 to 2200 m in Switzerland. We estimated fledging dates from a nationwide citizen science bird monitoring dataset and used the date when the proportion of observations of ‘fledged young’ reached 50% among all observations indicating breeding behaviour. This measure had the advantage that we could estimate average timing of the broods across a wide geographic range and over many years without the need to search for individual nests. We then compared differences in timing of the broods with climatic conditions and larch budburst across different elevational bands. The daily mean air temperature of 10–15°C was reached 34–38 days later at 2200 m compared to 1500 m, which is a similar delay as found in previous reports on snow melt‐out date. The average delay in larch budburst was 19.2 days at 2200 m compared to 1500 m. In comparison, the average timing of the birds' broods was only 5.4 days later in coal tits and 0.5 days later in Alpine tits at 2200 compared to 1500 m (the two species for which we had the narrowest interval estimates). Also, the estimated delay at higher elevations in the broods of song thrushes, mistle thrushes and Eurasian chaffinches was relatively small. Rather than postponing breeding dates to better environmental conditions later in the season that would match the earlier conditions at low elevation, songbirds breeding at higher elevations may thus have evolved adaptations to cope with the harsher conditions.

## Graphical abstract

Climatic conditions and food availability change with increasing elevation, thus breeding phenology of song birds may be delayed at higher compared to lower elevation. To investigate differences in average timing of broods along an elevational gradient, we used data from a nationwide citizen science bird monitoring scheme within the Swiss Alps. The investigated bird species raised their young with a smaller delay at higher elevation than expected, thus under harsher environmental conditions than at lower elevations.

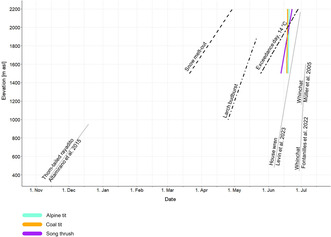

## 1 INTRODUCTION

Physical environmental factors such as temperature or snow melt‐out change with increasing elevation. In the Swiss Alps, for example, temperature decreases by an average of 0.65°C per 100 m increase in elevation (ISO 2533:1975), and mean snow melt‐out date is delayed by about 40 days at an elevational difference of 700 m (1500 vs 2200 m asl; Schano et al., 2021). Due to later snow melt‐out dates, harsher climatic conditions and resulting differences in food availability, the breeding season of birds is usually shorter at higher compared to lower elevations (Dillon & Conway, 2015; Labarbera & Lacey, 2018; Yeh & Price, 2004), and thus breeding at higher elevations is under time pressure (Illán et al., 2012; Stier et al., 2014 for butterflies).

At lower elevations, the timing of breeding in birds is often correlated with the peak of insect abundance in spring (Daan et al., 1989; Hinks et al., 2015; Lack, 1950; Visser & Both, 2005), and this peak is usually correlated with spring temperatures (Thackeray et al., 2016; Visser & Holleman, 2001; Vitasse et al., 2021). Accordingly, birds may fine‐tune their phenology to that of lower trophic levels; for example, in a lowland population of great tits (*Parus major*), there was a strong correlation between oak budburst (as a proxy for plant phenology) and the peak in caterpillar abundance, and oak phenology close to the nest was related to the timing of egg‐laying and hatching of the young (Hinks et al., 2015). The (possibly indirect) relationship between bird breeding phenology and spring temperatures was investigated by Verhagen et al. (2020) who found that great tits started breeding earlier when exposed to warmer compared to colder temperatures in climate‐controlled aviaries. Similarly, Saracco et al. (2018) found a difference of 11–14 days in mean capture dates of the young of 25 North American bird species between the warmest and coldest spring in their study.

Cold temperatures and late snow melt‐out date can thus delay the timing of breeding in birds (e.g. Pereyra, 2011; Smith & Andersen, 2014; Williams, 2012). Accordingly, previous studies found a delayed breeding phenology of songbirds at higher compared to lower elevations (Table 1). For example, Perfito et al. (2004) investigated changes in the testis volume of adult male song sparrows (*Melospiza melodia morphna*) and found a delay in testis growth of 1–2 months between coastal (0–10 m) and montane (280–1220 m) habitats. Further, Johnson et al. (2018) found that tree swallows (*Tachycineta bicolor*) began egg‐laying 10 days later at 2482 m (±41 m, SD) compared to 1359 m (±99 m), and Gil‐Delgado et al. (1992) found a delay of 7 days in the mean egg‐laying date for blue tits (*Cyanistes caeruleus*) at 900–1000 m compared to 500–750 m. Most of the studies on elevational differences in the timing of breeding investigated the start of egg‐laying of single populations by observing nests in the field (Table 1). While birds at higher elevations generally breed later, it is unclear whether this delay in reproduction allows them to experience the same optimal environmental and ecological conditions as birds that breed earlier at low elevations.

**TABLE 1:** List of different studies investigating timing of breeding along elevational gradients.

Species	Region	References	Observation	Low elevation	High elevation	Elevational difference	Low elevation			High elevation			Egg‐laying date				Fledging				Comments
							*N*	SD	SE	*N*	SD	SE	Low elevation	High elevation	Difference in days high versus low	Delay in days/100 m	Low elevation	High elevation	Difference in days high versus low	Delay in days/100 m	
Alpine tit Parus *montanus montanus*	Switzerland	Paterno et al.	Citizen science data observations	1500	2200	700											169.7 [164.2; 175.3]	170.9 [153.3; 182.3]	+1.1 [−19.8; +15.9]	0.19	
Carrion Crow *Corvus corone*	UK	Driver (2005)	Nest search	75	646	571	61			17			13 April–15 April	22 April–24 April	+9	1.58					
Coal Tit *Periparus ater*	Germany	Zang (1980)	Nest boxes	450	920	470										1.68					
	France	Bison et al (2020)	Nest boxes	1324	1874	550	60 nest boxes			50 nest boxes						1.5					A total quantity of 10%–45% of nest boxes were occupied in different years and elevations
		Clouet (2005)	Nest boxes	1325	1900	575	Nest boxes were occupied from 0 to 30%			37 nest boxes Nest boxes were occupied in max 55%			First = 19 April Mean = 5 May	First = 30 April Mean = 15 May	+10	First = 1.91 Mean = 1.74					No information on the number of nest boxes at low elevation
	Switzerland	Paterno et al.	Citizen science data observations	1500	2200	700											163.2 [157.9;169.0]	173.4 [161.8; 183.2]	+10.5 [−2.2; +20.6]	1.48	
Common Whitethroat *Curruca communis*	Germany	Bairlein et al. (1980)	Nest record cards	<250	>500		47			38			17 May	26 May	+9						Three elevational belts: <250, 250–500 and >500 m. Unclear at which elevation the gradient ends
Eurasian Blackcap *Sylvia atricapilla*	Germany	Bairlein et al. (1980)	Nest record cards	<250	>500		224			129			14 May	20 May	+6					Incubation and nestling period increase with increasing elevation	Three elevational belts: <250, 250–500 and >500 m. Unclear at which elevation the gradient ends
Eurasian Blue Tit *Cyanistes caeruleus*	Spain	Gil‐Delgado et al. (1992)	Nest boxes	625	950	325	25	7.25		83	8.43		6 May	13 May	+7	2.15					
	Germany	Zang (1980, 1982)	Nest boxes	120	540	420										Overall 5.24 Birds that are older than 1 year 5.37 1‐year‐old birds 4.17					
	France	Brändli (1998)	Nest boxes	532	1181	649	353	7		74	15		First = 27 March Mean = 13 April	First = 27 March Mean = 24 April	+11	First = 0 Mean = 1.69					
Eurasian chaffinch *Fringilla coelebs*	Switzerland	Paterno et al.	Citizen science data observations	1500	2200	700											174.8 [166.2; 182.8]	193 [156.3; 241.3]	+19.6 [−22.6; +68.5]	2.80	
European Pied Flycatcher *Ficedula hypoleuca*	Germany	Zang (1980)	Nest boxes	100	900	800										1.7					
Garden Warbler *Sylvia borin*	Germany	Bairlein et al. (1980)	Nest record cards	<250	>500		126			221			22 May	31 May	+9					Incubation and nestling period increase with increasing elevation	Three elevational belts: <250, 250–500 and >500 m. Unclear at which elevation the gradient ends
Great Tit *Parus major*	Germany	Zang (1980, 1982)	Nest boxes	100	900	800										Overall 2.19 Birds that are older than 1 year 2.13 1‐year‐old birds 2.45					
	Austria	Schöll and Hille (2014)	Nest boxes	544	789	245	35			21			13 Apr	20 Apr	+7	2.86					
Grey Wagtail *Motacilla cinerea*	UK	Ormerod & Tyler (1987)	Nest search	75	370	295										First‐egg dates ~13 days/100 m					The number of observations is unclear, since additional data were used for this particular analysis
Grey‐backed Shrike *Lanius tephronotus*	Tibet	Lu et al. (2010)	Nest search													Later					
House Wren *Troglodytes aedon*	California	Levin et al. (2023)	Nest boxes	480	2164	1684	83 nest boxes		4–6.5	73 nest boxes		2–.8	1 April–9 June	20 May–11 June	+26	1.54	6 June	30 June		1.54	No information about how many nest boxes were occupied
Lesser Whitethroat *Curruca curruca*	Germany	Bairlein et al. (1980)	Nest record cards	<500	>500		31			51			17 May	22 May	+5						
Marsh Tit *Poecile palustris*	Germany	Zang (1980)	Nest boxes	120	540	420										1.97					
Mistle thrush *Turdus viscivorus*	Switzerland	Paterno et al.	Citizen science data observations	1500	2200	700											188.0 [−134.6; 479.4]	187.6 [172.7; 214.4]	+32.8 [−295.7; +329.4]	4.69	
Mountain Bluebird *Sialia currucoides*	Wyoming	Johnson et al. (2006)	Nest boxes	1482	2504	1022	2004: 30 + 33, 2005: 18 nest boxes			2004: 68, 2005: 112 nest boxes			26 Apr	10 May	+14	1.37					No information about how many nest boxes were occupied
Northern Raven *Corvus corax*	France, Corsica	Delestrade (2002)	Nest search	20	1500	1480										Later					
Oregon Juncos *Juncus hyemalis oregonus*	Canada	Bears (1999)	Nest search	1021	1985	964	72			45			17 April–5 August	27 May–15 June	‐6	First = 4.15					
Pacific Wren *Troglodytes pacificu*s	Canada	Ogden et al. (2012)	Nest search	200	1010	810	40			6			ca. 21 April	ca. 31 May	+40	4.93					
Red‐billed Chough *Pyrrhocorax pyrrhocorax*	Spain	Blanco et al. (1998)	Nest search	330	1300	970	33	6.8		60	10.9		24 March	04 Apr	+11	1.13					
Red‐faced Warbler *Cardellina rubrifrons*	Arizona	Dillon and Conway (2015)	Nest search	1964	2711	747										1.82					
Rock Sparrow *Petronia*	Italy	Tavecchia et al. (2002)	Nest boxes	1570	1780	210	79			65			22 June	27 June	+5	2.38					
Russet Sparrow *Passer cinnamomeus former rutilans*	China	Yang et al (2012)	Nest boxes	200	1500	1300	24	15		57	19		9 May	7 June	+29	2.23					
Small Ground Finch *Geospiza fuliginosa*	Galapagos Archipelago	Kleindorfer (2007)	Nest search	50	500	450	90			63			27 February	2 February	−25	−5.56					
Song Sparrow *Melospiza melodia*	Washington State	Perfito et al. (2004)	Mist nets	5	750	745	11			10			April	May/June							
Song Thrush *Turdus philomelos*	Switzerland	Paterno et al.	Citizen science data observations	1500	2200	700											168.7 [119.7; 215.3]	169.6 [163.2; 175.3]	+1.8 [−47.2; +49.5]	0.26	
Thorn‐tailed Rayadito *Aphrastura spinicauda*	Chile	Altamirano et al. (2015)	Nest boxes direct observations camera traps	368	952	584	High and low together = 103			High and low together = 103			7 October	12 November	+36	6.16	13 November	19 December	+36	6.16	
Tree Swallow *Tachycineta bicolo*r	Wyoming	Johnson et al. (2018)	Nest boxes	1359	2482	1123	80			140			31 May	10 June	+10	0.89					In total, 28 females were sampled in both years and not all data were obtained for each female and clutch in each year, so sample sizes vary for different analyses
Whinchat *Saxicola rubetra*	Switzerland	Müller et al. (2005)	Nest search	1160	1600	440	12			44			29 May	3 June	+5	1.31	1 July	5 July	+4	1.14	
	France	Fontanilles (2022)	Nest search	440	1468	1028	10			30							24 June–5 July	22 June–16 July	+13	0.58	
White‐bellied Redstart *Luscinia phaenicuroides*	Tibet	Lu et al. (2010)	Nest search	1325	4325	3000	38		4	18		4	9 June	19 June	+10	0.33	2 July	21 July	+19	0.63	Fledging dates at low elevation ranged from 7 May to 9 August and at high elevation from 31 May to 9 August
White‐throated Dipper *Cinclus cinclus*	Scotland	Logie (1998)	Nest search	106.08 + − 64.80	305.75 + − 97.56	200	305			103			8–10 March	17–19 March	+9	4.5					

Several previous studies also investigated national or international datasets and used data from bird nest record cards to deduce the start of breeding (Bairlein et al., 1980; Dunn et al., 2000; Gibbs, 2007). For example, Bairlein et al. (1980) investigated elevational differences in the start of egg‐laying of four *Sylvia* species, using more than 2000 nest record cards from Germany, Finland, France and the Canary Islands; they found a delay in egg‐laying date of 5–9 days between the highest and lowest elevational belt (<250 m and >500 m) depending on the species. The interpretation of the delay is difficult, since Bairlein et al. (1980) investigated three elevational belts (including 250–500 m) and it is unclear where the elevational gradient ended. Another study also used data from nest record cards: Gibbs (2007) found a delay in breeding of Australian Magpies (*Gymnorhina tibicen*) of 2.7–3.9 days per 100 m increase in elevation, depending on the dataset used. Gibbs (2007) investigated data from different databases like the Australian Nest Record Scheme (NRS), the Garden Bird Survey of the Canberra Ornithologists (GBS), the Atlas of Australian Birds and the NSW Bird Atlas to estimate hatching and fledging dates of Australian Magpies. The NRS and the GBS represented suitable sources for their study because detailed breeding data were available. In contrast, only little information on the timing of breeding was available in the Atlas of Australian Birds and the NSW Bird Atlas (Gibbs, 2007). Thus, nationwide datasets can be used to investigate the timing of breeding in birds, but suitability of a dataset depends on how information on the timing of the brood is recorded.

Several nationwide or continent‐wide citizen science breeding bird monitoring schemes are primarily used to gain information on population trends and ranges (e.g. Keller et al., 2020; Knaus et al., 2018). Because such nationwide bird monitoring schemes may not involve systematic nest searches, the resulting data often may not be suitable to directly deduce egg‐laying or fledging dates. However, there may be sufficient information to estimate the timing of the broods if the observed bird behaviour is recorded in a standardised way such as using international breeding codes (e.g. Keller et al., 2020).

**FIGURE 1:**
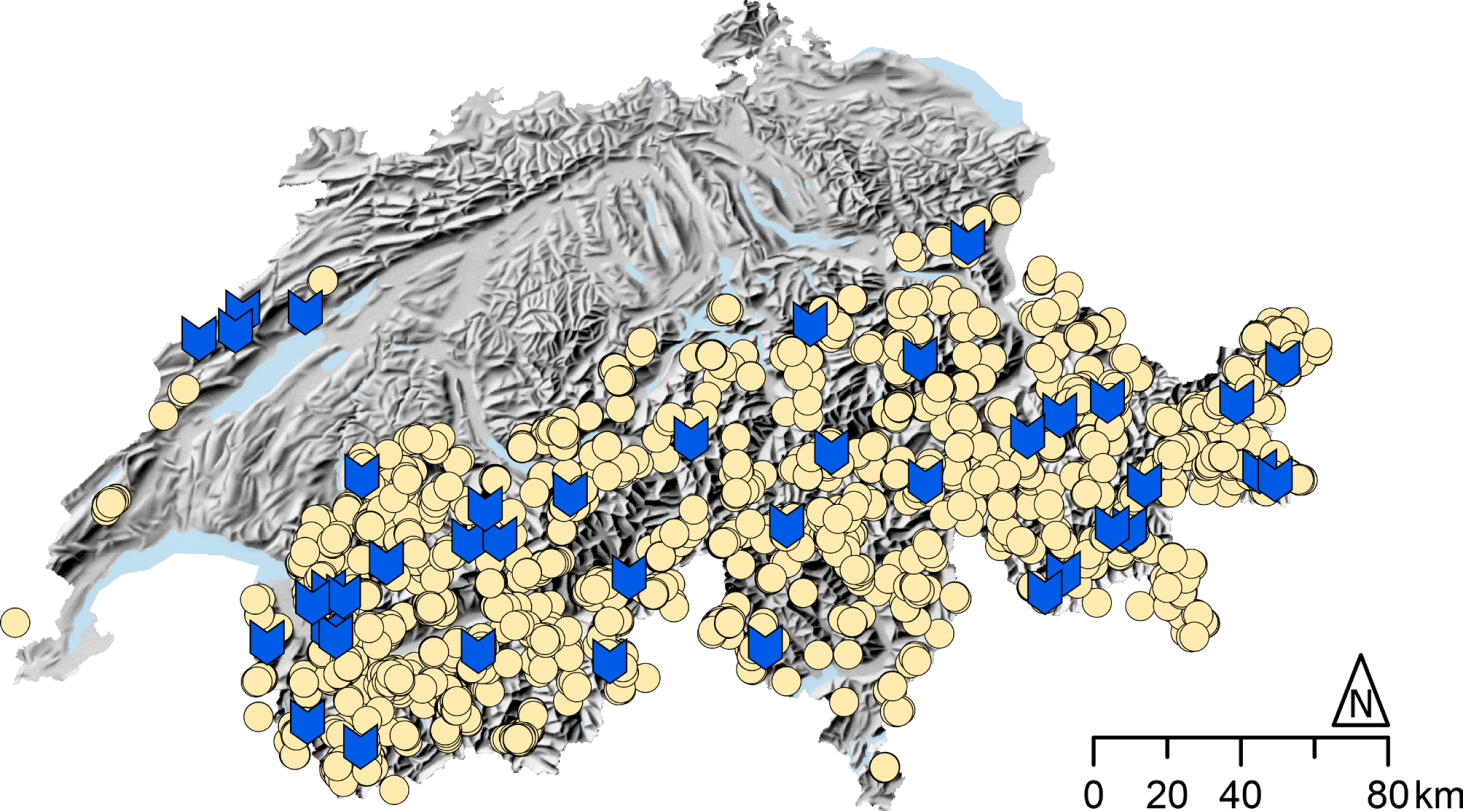
Map of Switzerland, with yellow dots indicating bird observations used for this study, and blue marks indicating the 41 Swiss Phenology stations of MeteoSwiss that were used to calculate the date of larch budburst at different elevations.

In this study, we used data from the Swiss monitoring of common breeding birds (Knaus et al., 2018) and opportunistic observations from the online database ornitho.ch (Ornitho, 2023; data obtained from the Swiss Ornithological Institute; Figure 1, Tables 2 and 3) to investigate the breeding phenology of five woodland songbird species all over Switzerland along an elevational gradient from 1500 to 2200 m. We estimated the dates when the fledged young were reported in 50% of the observations that indicated a brood, which means that no nest searches and thus no disturbance at the nests were necessary. We also compared climatic conditions and larch budburst between 1500 and 2200 m. Since previous studies found strong correlations between plant phenology and food availability (e.g. Hinks et al., 2015), we used larch budburst as a proxy for food availability. The European larch (*Larix decidua*) is a suitable species to investigate plant phenology in our study because the main distributional area of the European larch is between 1500 and 2000 m (up to 2250 m; Brändli, 1998), and measurement of larch budburst is a common proxy for plant phenology at higher elevations (Brügger & Vasella, 2003). We expected a later rise of spring temperatures, later snow melt‐out dates and a delay in larch budburst at higher elevations; accordingly, we expected later observations of fledged young birds at 2200 m compared to 1500 m.

**TABLE 2:** Atlas codes describing observations of a brood in birds (Schweizerische Vogelwarte Sempach, 2019), the developmental stage associated with the specific Atlas code, category in relation to fledging, assigned response category for the analyses (Bernoulli model) and the number of observations per Atlas code.

Atlas code		Developmental stage	Category	Value of response variable	Number of observations
9	Female adult with breeding spot	Incubation or nestlings	Certainly before fledging	0	4
15	Adult carrying faecal sac from the nest	Nestlings	Certainly before fledging	0	5
16	Adult with food for nestlings	Nestlings (or freshly fledged)	Likely before fledging, but after fledging possible	0	853
18	Nest with breeding adult	Incubation or nestlings	Certainly before fledging	0	72
19	Nest with eggs or nestlings	Incubation or nestlings	Certainly before fledging	0	26
13	Recently fledged birds	Fledged	Certainly after fledging	1	1020

**TABLE 3:** Number of observations per species and year.

Year	Coal tit	Alpine/willow tit	Eurasian chaffinch	Song thrush	Mistle thrush
2013	38	30	11	11	50
2014	114	54	23	16	48
2015	147	44	36	21	59
2016	97	30	54	59	91
2017	52	11	12	5	33
2018	51	20	24	14	35
2019	57	12	22	20	45
2020	52	18	28	21	58
2021	73	13	30	8	43
2022	65	26	24	17	58
Total	764	258	264	192	520

## 2 MATERIALS AND METHODS

All data used in this study were collected throughout the mountainous regions of Switzerland. Larch budburst data were collected across the same elevations and in close proximity to the breeding phenology observations, but not at the exact locations of the breeding bird observations (Figure 1). Data on temperature covered the elevational range between 1500 m and 2200 m of the whole country (i.e. a similar region as for bird and larch budburst observations). The elevational range for data on temperature and breeding phenology was the same (1500–2200 m); for larch budburst, we used a slightly lower elevational range because data were only available up to 1933 m (i.e. there were no Swiss Phenology stations of MeteoSwiss above 1933 m), and we predicted larch budburst for the higher elevations (see below for further explanation).

### 2.1 Temperature and larch budburst

**FIGURE 2:**
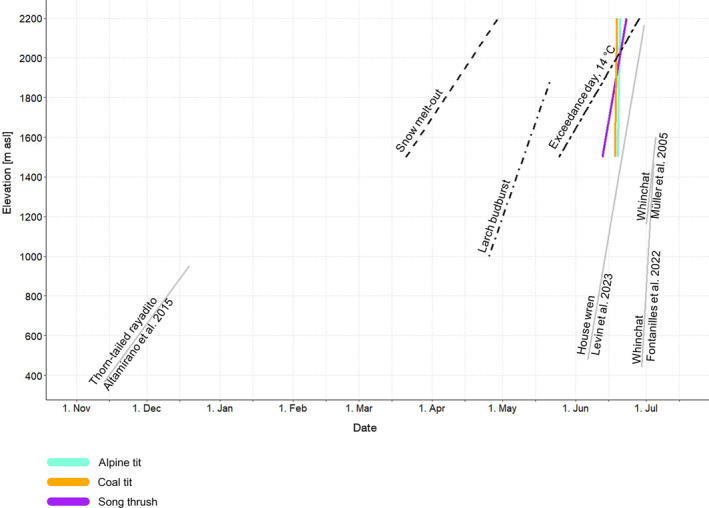
Coloured solid lines show the dates when the broods of coal tit, Alpine tit and song thrush became certainly fledged in 50% of observations in an average year (in this plot, we used only the species and elevations for which estimates had sufficient precision). Dotted lines show the date of mean snow melt‐out, based on data from Schano et al. (2021); the date when a temperature threshold of 14°C was exceeded (Figure 3); and larch budburst, defined as the date when 50% of the young tufts of needles of a single tree or tree stand began to loosen up and spread (Brügger & Vasella, 2003). Grey lines show the mean dates when young of three other songbird species fledged based on data from the literature (Altamirano et al., 2015; Fontanilles, 2022; Levin et al., 2023; Müller et al., 2005, see also Table 1).

To compare the timing of the warm‐up between 1500 m and 2200 m, we used the dates when the mean daily temperature exceeded thresholds between −5 and +15°C (mean daily temperatures above 15°C are rarely reached at 2200 m), in steps of 1°C, at 1500 m and 2200 m throughout Switzerland. This analysis was based on the daily mean air temperature grid at 2 × 2 km resolution of Switzerland, that is, on the TabsD dataset for the years 1991–2022 by MeteoSwiss (Frei, 2013). For each year and temperature threshold, the first day of the year (in the following called exceedance day) on which the respective temperature threshold was exceeded in spring or early summer was determined. Each exceedance day was calculated using a stepwise procedure for each temperature threshold separately. First, we calculated the exceedance day for a particular threshold at each of the grid cells of the countrywide dataset for each year separately. We then determined the median exceedance day on each of the two elevational bands separately. The elevational bands are defined as all grid cells within ±100 m around 1500 m and 2200 m, respectively. We calculated the difference of the exceedance days between the two elevational bands for each year, and our final result was the median difference in exceedance days of all years between 1991 and 2022 per temperature threshold. To investigate differences in plant phenology, we calculated differences in larch budburst between 1500 m and 2200 m using data from MeteoSwiss. Data were available from 2013 to 2022 from 41 sites (Swiss Phenology Network) distributed over Switzerland between 1000 and 1933 m (Figures 1 and 2). For those sites, we extracted the date when 50% of the young tufts of needles of a single tree or tree stand began to loosen up and spread (= larch budburst, Brügger & Vasella, 2003). We used the R base package (R Core Team, 2020) to build a linear model on the date of larch budburst as predicted by the two continuous variables elevation and year, and used the regression line of best fit to predict the expected difference in number of days of average larch budburst between 1500 m and 2200 m, since data were only available up to 1933 m.

### 2.2 Estimation of the timing of the broods

We analysed data on five common woodland bird species that breed across a large elevational range in Switzerland: song thrush (*Turdus philomelos*), mistle thrush (*Turdus viscivorus*), Eurasian chaffinch (*Fringilla coelebs*), coal tit (*Periparus ater*) and Alpine (*Poecile montanus montanus*) or willow tit (*Poecile montanus rhenanus/*salicarius; Table 3). In Switzerland, both *Poecile montanus* forms exist, occupying different ecological niches. The Alpine tit can be found in higher regions, especially in the Alps, whereas the willow tit is more frequent in the lowlands (Glutz von Blotzheim & Bauer, 1993; Knaus et al., 2018). We summarised all observations as Alpine tits because morphological differentiation between the two forms in the field is difficult, and because the elevational range considered in this study starts at the upper range of the willow tit distribution and covers the main distribution of the Alpine tit (Knaus et al., 2018).

For estimation of the timing of the brood (as defined below), we used data from the Swiss monitoring of common breeding birds (Knaus et al., 2018), which is part of the Swiss Biodiversity Monitoring program (BDM Coordination Office, 2014; data obtained from the Swiss Ornithological Institute; Figure 1, Tables 2 and 3). We additionally used opportunistic observations from the online database ornitho.ch (Ornitho, 2023; data obtained from the Swiss Ornithological Institute; Figure 1, Tables 2 and 3) that were collected between 2013 and 2022 (before 2013, there were not enough data available). Both datasets are mostly collected by volunteer naturalists and thus are from nationwide citizen science projects.

**FIGURE A1:**
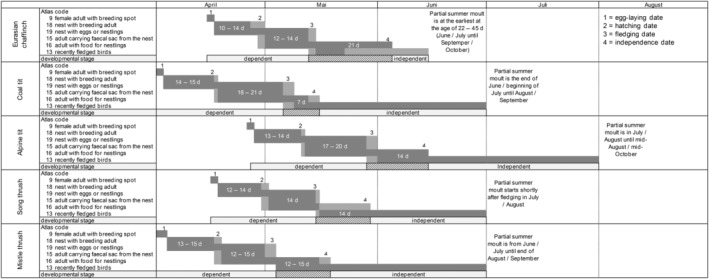
Timeline of breeding dates (data obtained from Bauer et al., 2012), Atlas code descriptions and the developmental stage assigned for the five investigated songbird species. The dark grey shading shows the minimum time, the light grey shading the maximum time needed for a certain breeding stage. The lined area indicates the time period where the two developmental stages in our study overlap.

The combined dataset included records on species, date, coordinates, elevation and the ‘Atlas code’ providing descriptions of bird behaviour and specifying whether a brood is either possible, likely or certain; the Atlas codes used in Switzerland (Knaus et al., 2018; Swiss Ornithological Institute, 2019) are based on the international Atlas codes (Keller et al., 2020). In the online database ornitho.ch, the Atlas codes are required for entries of all breeding birds in Switzerland during the breeding season (Ornitho, 2023). We selected observations that indicated a brood and that allowed to distinguish between early and late stages of the brood (Tables 2 and S1). For each breeding observation, we thus assigned one of the two developmental stages ‘early; certainly or likely not fledged’ and ‘late; certainly fledged’ and then calculated the proportion of broods with certainly fledged young among all observed broods at a given elevation. We included the observation ‘adults with food for their young’ in the early stage even though in the investigated species adults continue feeding their young for a few days up to 2–3 weeks after fledging (Figure A1; Glutz von Blotzheim, 1988, 1997; Glutz von Blotzheim & Bauer, 1993). Eurasian chaffinch fledglings are relatively independent after about 1 week, but occasional feeding by adults can occur up to 3 weeks after fledging (Glutz von Blotzheim, 1997); and Alpine tits become independent about 5–6 days after fledging, with occasional feeding events up to 2 weeks after fledging (Glutz von Blotzheim & Bauer, 1993). Because feeding frequency decreases after fledging (e.g. mistle thrush chicks are fed up to six times more often during the nestling period than after fledging; Glutz von Blotzheim, 1988), we assume that a record ‘adults with food for their young’ refers to the nestling period (early breeding stage) in the majority of the cases. However, a substantial portion of the Atlas code 16 observations may actually include fledged broods, because observers are instructed to record the highest Atlas code when more than one behaviour is observed. Therefore, ‘adults with food for their young’ (Atlas code 16) instead of ‘freshly fledged young’ (Atlas code 13) is usually recorded in cases when adults with food and fledged young are observed simultaneously.

As an alternative to estimating the dates when the fledged young were reported in 50% of the observations that indicated a brood from a combination of different Atlas codes, we could have used only the dates of observations of ‘recently fledged birds’ (Atlas code 13) to calculate average dates of fledging; in this case, however, results would have been strongly influenced by observer effort: Early in the season, there are probably fewer observers present at higher elevations, and thus average fledging dates at higher elevations would have been biased towards a later date simply because there are fewer early observations available.

### 2.3 Statistical analysis of timing of the broods

We used R version 4.0.3 (R Core Team, 2020) and the package birdring (Korner‐Nievergelt & Robinson, 2019) to prepare the data. We used a separate binomial generalized linear model (GLM) to estimate the proportion of young birds observed as fledged in relation to the two continuous variables date (day of year) and elevation for each year. To do so, we assigned to each observation whether young were certainly fledged or not (a binary response variable *y*; Table 2). We then used elevation, date and a categorical variable ‘year’ as predictors. We included a two‐way interaction of elevation and date. To account for among‐year differences in the onset of spring, we included a random year effect *d*_year_ (i.e. the difference between the timing of the brood in a given year and the average timing from all years) on the predictor variable ‘date’ (day of year). Thus, we assumed that the strength of the increase of the proportion of independent young birds (*p*) with increasing date as well as its elevational gradient is equal across years, but we allowed the average timing of the broods to vary among years according to a normal distribution:

$$y_{i}\sim \text{Bernoulli}\left(p_{i}\right)$$

$$\begin{array}{c} \text{logit}\left(p_{i}\right)=\beta_{0}+\beta_{1}{\text{elevation}}_{i}+\beta_{2}\left({\text{date}}_{i}+d_{\text{year}\left[i\right]}\right)\\ \;+\beta_{3}\left({\text{date}}_{i}+d_{\text{year}\left[i\right]}\right){\text{elevation}}_{i} \end{array}$$

$$d_{\text{year}}\sim \text{Normal}\left(0,\sigma \right)$$

We thus used a logit‐link function and assumed a Bernoulli distribution. We also assumed a sigmoid increase of the proportion of certainly fledged young birds among the observed broods over the breeding season, and we allowed for a gradual change in slope with elevation, that is, we allowed for steeper increase in the certainly fledged young as would be expected when breeding season is shorter at higher elevations. Elevation and day of the year were z‐transformed. We fitted the model in Stan (http://mc‐stan.org, Carpenter et al., 2017) via the interface rstan (Stan Development Team, 2023) using Hamiltonian Monte Carlo (Betancourt, 2013). The model code is available in Data S1. We simulated four chains of length 2000 and used the second half of each, that is, we used a total 4000 draws to describe the posterior distributions of the model parameters. We thus present the difference in the number of days of the date when 50% of the broods were certainly fledged between elevations as well as the 2.5% and 97.5% quantiles as lower and upper limits of the 95% Bayesian compatibility intervals (Amrhein & Greenland, 2022). We present the results for the three elevations low = 1650 m, medium = 1850 m and high = 2050 m, to show the change in proportion of certainly fledged young birds as the season progresses.

## 3 RESULTS

### 3.1 Temperature and larch budburst

**FIGURE 3:**
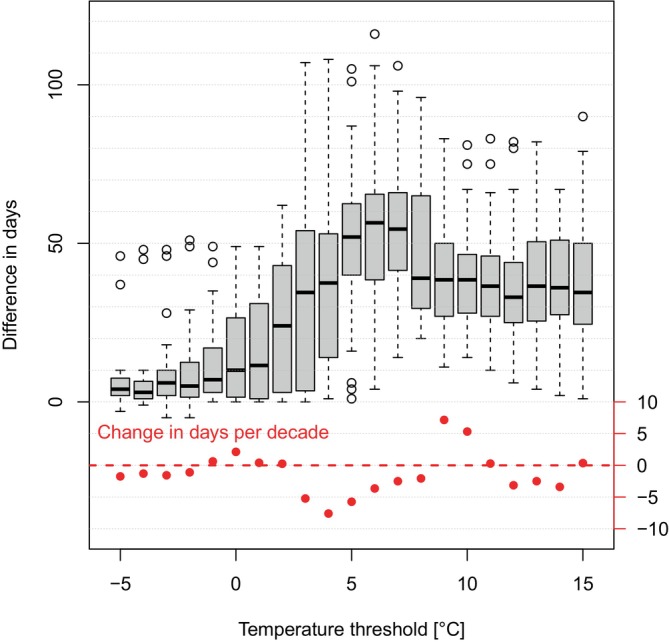
Difference in the number of days that a certain temperature threshold was reached at 2200 m compared to 1500 m. Data are based on the dataset TabsD (years 1991–2020) by MeteoSwiss (Frei, 2013). Red data points show the change in number of days per decade over the last 30 years.

In spring, temperature thresholds below 0°C were exceeded at about the same time at 1500 m and 2200 m. With increasing temperature (0–7°C), the difference in exceedance days between 1500 m and 2200 m increased, and daily mean air temperatures of 10–15°C were reached, on average, 34–38 days later at 2200 m compared to 1500 m (Figures 3 and S1). However, these differences in the exceedance days between the two elevational bands 2200 m and 1500 m showed a high interannual variability. For temperature thresholds between 10°C and 15°C, the difference ranged between 5 days and approximately 70 days in individual years. A clear trend indicating a smaller or larger difference in the exceedance day between the two elevations was not evident; thus, we found no indication that climate change was accelerating warming more rapidly at one elevation versus the other. For example, the difference in the exceedance days increased by 5 days per decade for a temperature threshold of 10°C, by 0 days per decade for a 11°C and decreased by −3 days per decade for a temperature threshold of 12°C (Figure 3).

Between 2013 and 2022, larch budburst in Switzerland started on average 19.2 days later at 2200 m compared to 1500 m. Larch budburst, therefore, was delayed by 2.74 days per 100 m increase in elevation (Table 4).

**TABLE 4:** Parameter estimates and 95% CI for larch budburst in relation to elevation and year.

	Standardised coefficients mean [95% CI] in days	Standard deviation of the predictor variable	Unstandardized coefficients mean [95% CI] in days	Unit of the predictor variable
Intercept	+904.48 ± 430.10			
Elevation	+0.03 ± 0.00	243 m	+0.01 ± 0.00	100 m
Year	−0.41 ± 0.21	2.9 days	−0.14 ± 0.07	1 day

### 3.2 Dates when 50% of the broods are certainly fledged

**FIGURE 4:**
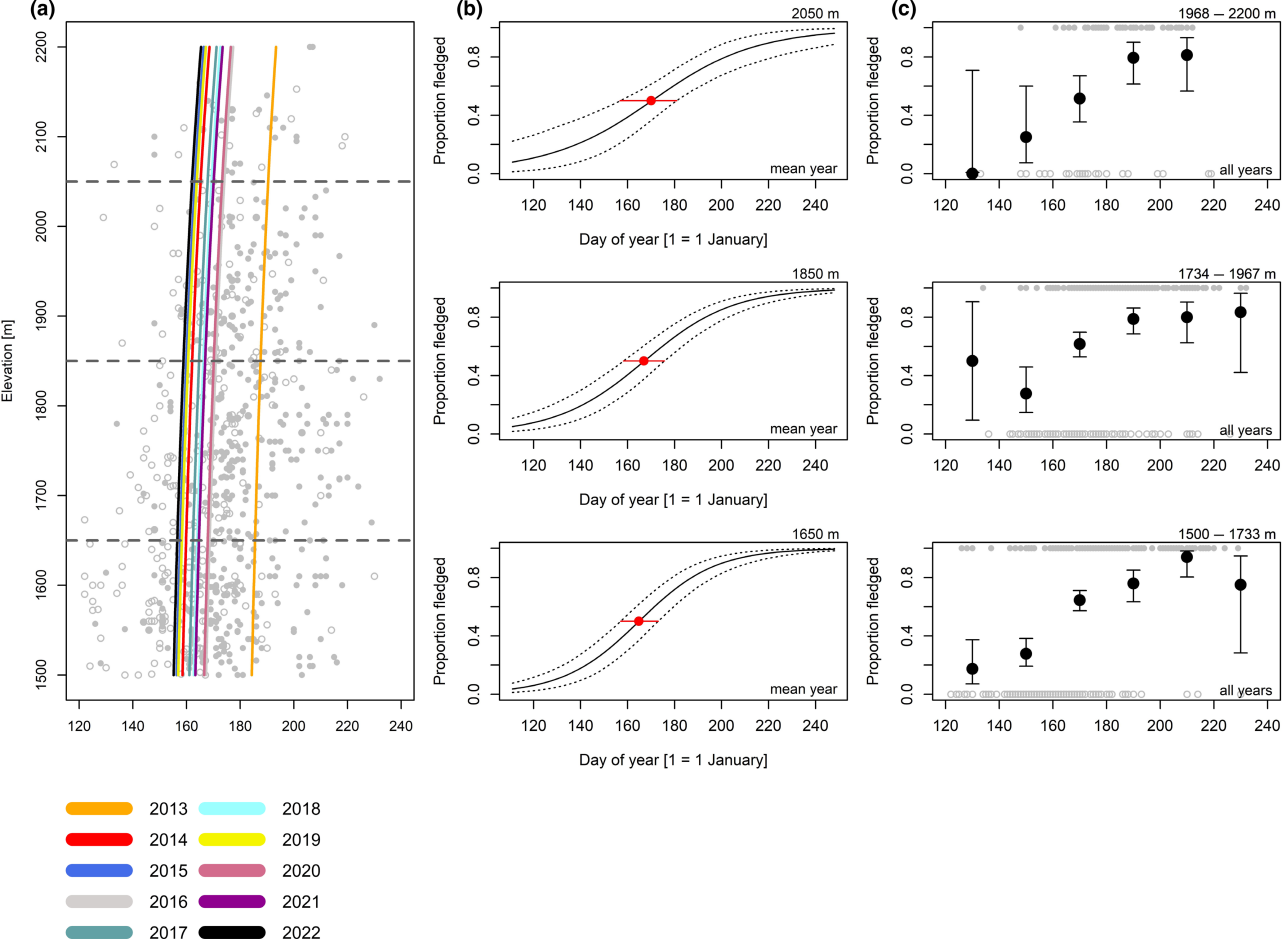
Coal tit. Grey dots are all observed broods (empty = certainly or likely not fledged, full = certainly fledged; for our definition of those stages, see main text). Lines in (a) show the dates when the broods were certainly fledged in 50% of observations from a given year (for a more straightforward comparison, the uncertainty in our estimates is depicted in the plots (b)). The horizontal grey dashed lines show the average elevations used in (b). Plots in (b) show the modelled proportions of observations from broods certainly or likely not fledged at 1650 m, 1850 m and 2050 m in an average year; dotted lines are 95% Bayesian compatibility intervals. Red dots show the dates when the broods became certainly fledged in 50% of observations of a brood in an average year; red lines are 95% Bayesian compatibility intervals. Plots in (c) show the proportions and 95% Bayesian compatibility intervals of certainly fledged observations, based on raw data, over 20‐day bins summarized for three elevational belts (1500–1733, 1734–1967 and 1968–2200 m). Note that what matters are the relative differences in dates among elevations. Estimates and 95% Bayesian compatibility intervals to plots in (a) and (b) are in Tables 5 and 6.

**FIGURE 5:**
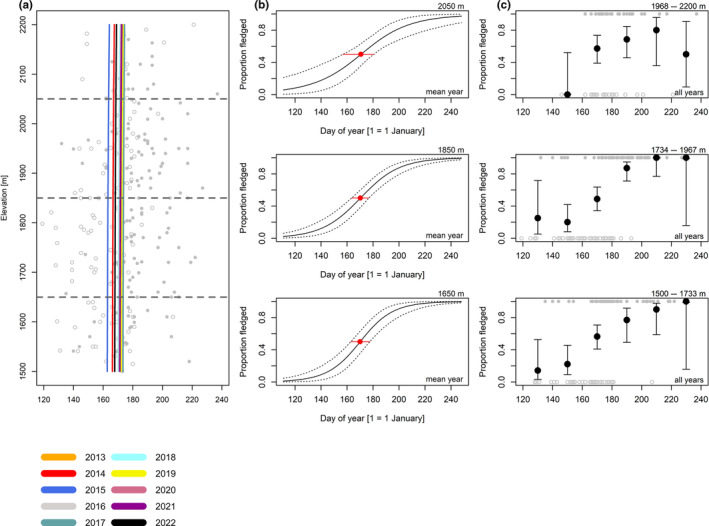
Alpine/willow tit; for explanation, see Figure 4.

**FIGURE 6:**
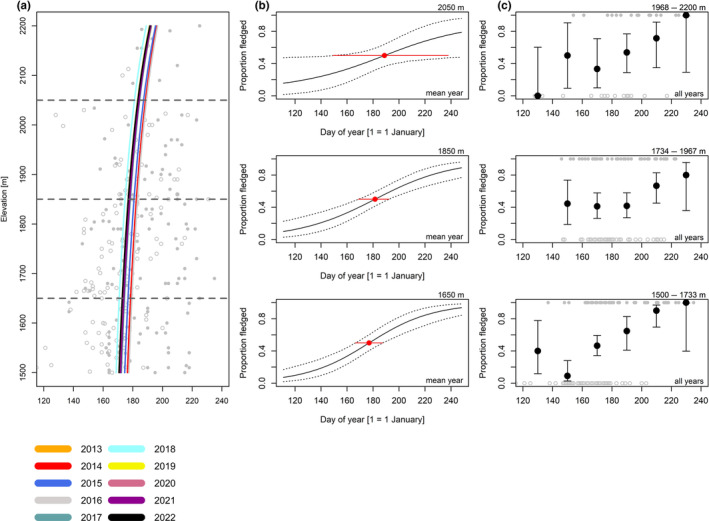
Eurasian chaffinch; for explanation, see Figure 4.

**FIGURE 7:**
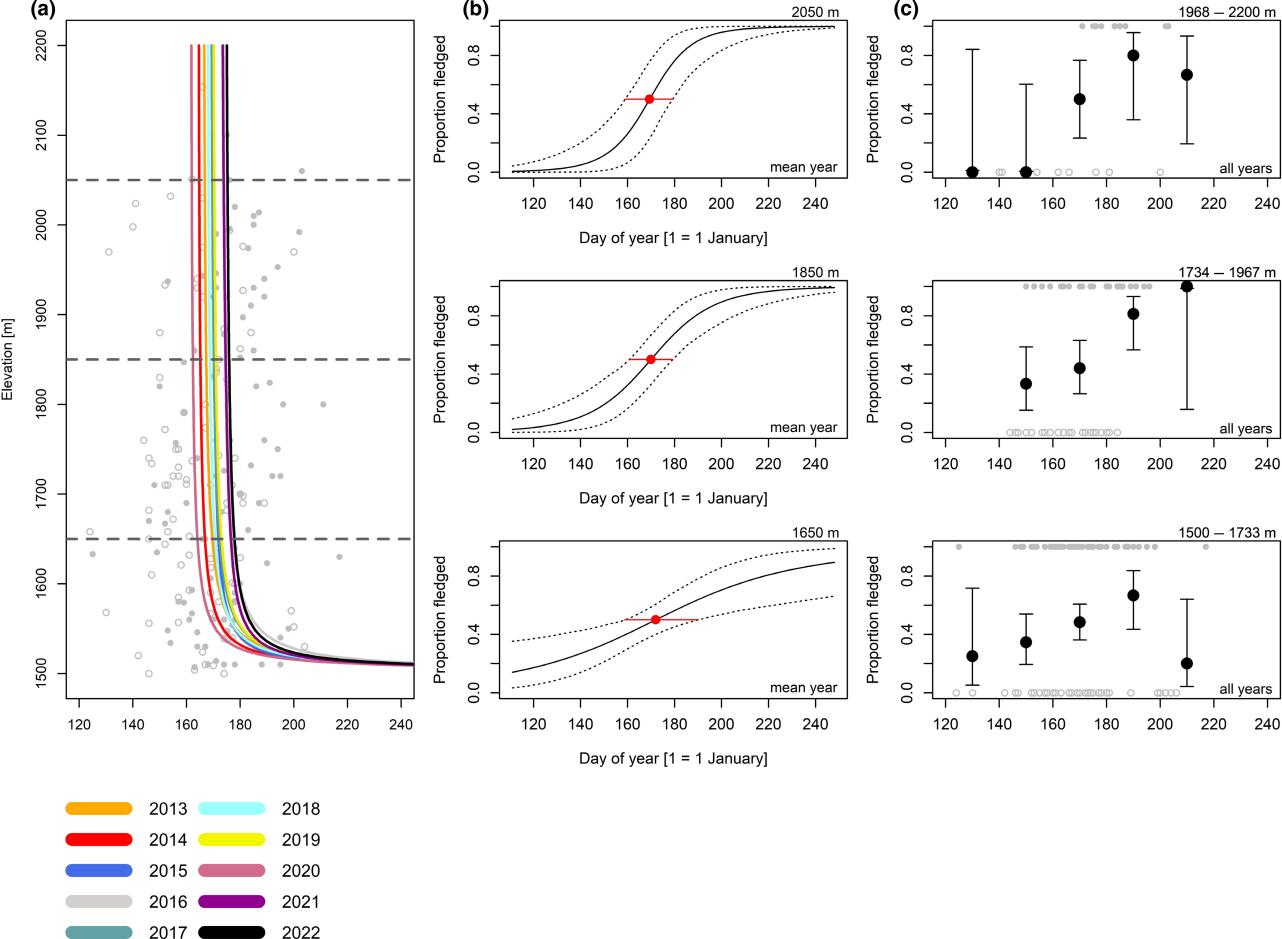
Song thrush; for explanation, see Figure 4.

**FIGURE 8:**
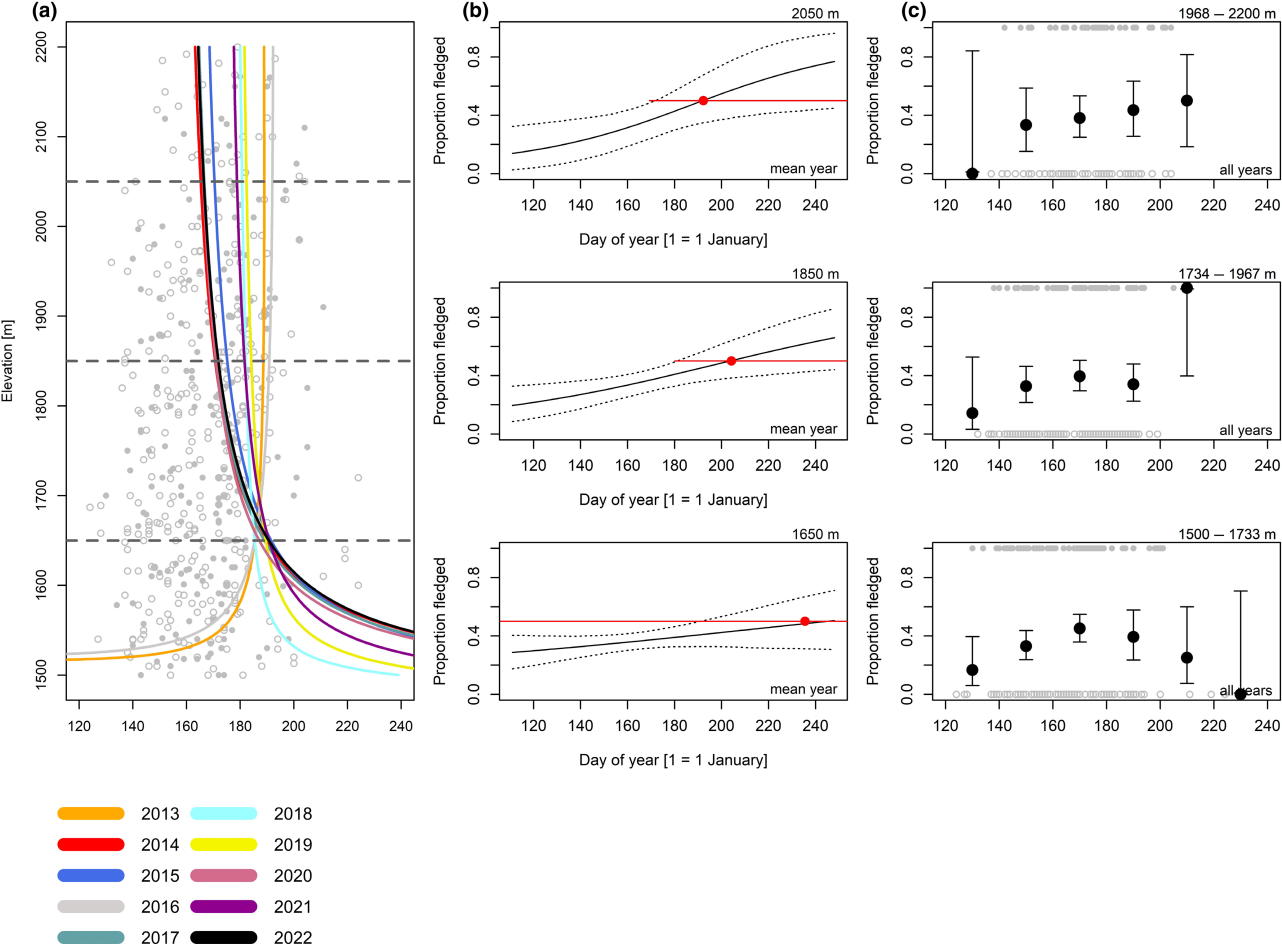
Mistle thrush; for explanation, see Figure 4.

Overall, we found strong variation from year to year in the dates when young broods certainly fledged (as opposed to ‘certainly or likely not fledged’) in 50% of observations (Figures 4, 5, 6, 7, 8, Table 5), with Eurasian chaffinches showing the least variation (Figure 6a, Table 5). However, we found that the date when broods were certainly fledged in 50% of observations was relatively similar between high and low elevation in most species. In the following, we compare the date when broods were certainly fledged in 50% of observations at three elevations (low = 1650, medium = 1850 and high = 2050; see also Figures 4, 5, 6, 7, 8, Table 6) in an average year. Due to increased uncertainty in the estimates at high elevation, a comparison between high and low elevation was not possible for all species, so in some cases, we used the medium elevation for comparison with the low elevation.

**TABLE 5:** Estimates to Figures 3, 4, 5, 6, 7. Estimates and 95% Bayesian compatibility intervals (CI; Amrhein & Greenland, 2022) in number of days, in relation to species and year for the mean elevation (1743 m).

Year	Coal tit	Alpine / willow tit	Eurasian chaffinch	Song thrush	Mistle thrush
2013	−1.04 [−1.85; −0.38]	−0.04 [−0.55; +0.44]	+0.01 [−0.77; +0.93]	+0.08 [−0.62; +0.92]	−0.98 [−4.10; +0.57]
2014	+0.24 [−0.23; +0.76]	+0.15 [−0.21; +0.68]	−0.13 [−1.26; +0.36]	+0.18 [−0.34; +1.15]	+0.67 [−0.75; +3.05]
2015	+0.35 [−0.09; +0.84]	+0.32 [−0.07; +1.00]	−0.07 [−0.97; +0.46]	−0.02 [−0.70; +0.70]	+0.37 [−1.14; +2.67]
2016	−0.19 [−0.70; +0.34]	−0.09 [−0.64; +0.35]	−0.18 [−1.13; +0.25]	−0.20 [−0.94; +0.26]	−1.15 [−3.71; +0.32]
2017	+0.11 [−0.44; +0.69]	−0.13 [−0.95; +0.33]	+0.01 [−0.83; +0.82]	−0.02 [−0.90; +0.90]	+0.57 [−0.96; +2.94]
2018	+0.03 [−0.54; +0.66]	−0.01 [−0.58; +0.55]	+0.12 [−0.36; +1.34]	+0.03 [−0.74; +0.97]	−0.23 [−2.68; +1.67]
2019	+0.31 [−0.21; +0.94]	−0.09 [−0.74; +0.35]	+0.05 [−0.55; +0.98]	−0.03 [−0.68; +0.54]	−0.34 [−2.64; +1.23]
2020	−0.16 [−0.75; +0.41]	−0.04 [−0.58; +0.44]	+0.05 [−0.53; +0.88]	+0.31 [−0.20; +1.22]	+0.58 [−0.97; +2.98]
2021	+0.00 [−0.50; +0.51]	−0.06 [−0.70; +0.45]	+0.02 [−0.62; +0.76]	−0.15 [−1.35; +0.47]	−0.13 [−2.42; +1.79]
2022	+0.41 [−0.13; +1.01]	+0.08 [−0.34; +0.62]	+0.05 [−0.54; +0.84]	−0.23 [−1.13; +0.25]	+0.55 [−0.99; +3.07]

**TABLE 6:** Estimates to Figures 3, 4, 5, 6, 7. Estimates and 95% CI in days [1 = 1 January], in relation to the species and three different elevations for an average year.

Elevation	Coal tit	Alpine tit	Eurasian chaffinch	Song thrush	Mistle thrush
1650	164.9 [156.7; 173.3]	169.73 [162.8; 177.7]	177.1 [167.0; 187.2]	173.0 [159.4; 189.7]	237.8 [−343.7; 933.9]
1850	167.1 [158.3; 175.6]	170.02 [163.3; 177.1]	181.6 [169.2; 192.7]	170.1 [161.0; 179.4]	203.6 [179.0; 293.1]
2050	170.1 [157.1; 180.4]	170.45 [155.8; 180.3]	188.9 [153.3; 243.0]	169.6 [159.4; 179.1]	192.0 [167.0; 253.3]

For coal tits (Figure 4a, Table 5), the date when broods were certainly fledged in 50% of observations was 5.4 days later (1.48 days/100 m) at high compared to low elevation (Figure 4b, Table 6). For Alpine tits (Figure 5a, Table 5), the date when broods certainly fledged in 50% of observations was 0.53 days later (0.19 days/100 m) at high compared to low elevation (Figure 5b, Table 6). For Eurasian chaffinches (Figure 6a, Table 5), we found a delay of 4.5 days (2.86 days/100 m) in the date when broods were certainly fledged in 50% of observations at medium compared to low elevation, and the difference between high and medium elevation was similar (6.7 days) but had higher uncertainty, as shown by wider compatibility intervals (Figure 6b, Table 6). The date when song thrush broods were certainly fledged in 50% of observations was about the same at high compared to medium elevation (Figure 7a,b, Tables 5 and 6, 0.29 days/100 m). Nevertheless, we found later ‘certainly fledged’ observations at high compared to medium and low elevation (grey empty points in Figure 7c). The difference between the dates of the first ‘certainly fledged’ observations between the three elevations was smaller for mistle thrushes (5.06 days/100 m) than for song thrushes (grey points in Figures 7c and 8c). Song and mistle thrushes often have more than one brood per season, leading to high uncertainty in our estimates at all elevations especially for the mistle thrush (Figure 8b, Table 6). Additionally, there was a lack of observations of ‘certainly fledged’ broods for mistle thrushes at the end of the season at low elevations, when we still found observations of ‘certainly or likely not fledged’ broods (see grey points in Figure 8c); thus, it seems like mistle and song thrushes became ‘certainly fledged’ later at lower compared to higher elevations.

### 3.3 Comparison of environmental variables and fledging dates

The delay in snow melt‐out date across elevations (5.7 days/100 m; Schano et al., 2021) was similar to the delay in temperature exceedance days at 14°C (4.9–5.4 days/100 m; Figure 2). However, the delay in larch budburst timing across elevations was smaller (2.7 days/100 m), and the delay in dates when 50% of the broods were certainly fledged was even smaller (coal tits: 1.48 days/100 m, Alpine tits: 0.19 days/100 m, song thrushes: 0.29 days/100 m; Figure 2).

## 4 DISCUSSION

We found a similar delay in temperature threshold exceedance (daily mean air temperature 10–15°C) at higher elevations (a delay of 34–38 days at 2200 m compared to 1500 m, i.e. 4.9–5.4 days/100 m) as Schano et al. (2021) found for the mean snow melt‐out date (about 40 days delay at 2200 m compared to 1500 m, i.e. 5.7 days/100 m). The timing of larch budburst was delayed by an average of 2.7 days/100 m increase in elevation; thus, the delay was slightly shorter than in temperature and snow melt‐out.

Despite the harsher conditions at higher elevations earlier in the season, birds were raised with a relatively small delay at higher elevations: The mean delay in the time when broods were certainly fledged in 50% of observations was 1.48 days/100 m in coal tits and 0.06 days/100 m in Alpine tits, the two species for which we had the clearest results (the narrowest compatibility intervals). Therefore, the investigated songbird species were likely raised under harsher environmental conditions at higher elevations.

Previous studies reported similar elevational differences when investigating fledging dates (Table 1, Figure 2). For example, Müller et al. (2005) found an average delay in fledging date of 1.14 days/100 m for whinchats (*Saxicola rubetra*) in Switzerland, and Fontanilles (2022) found a delay of 0.58 days/100 m in the French Pyrenees. Lu et al. (2010) found a delay in fledging dates of 0.63 days/100 m for white‐bellied redstarts (*Hodgsonius phaenicuroides*) in Tibet, and Levin et al. (2023) a delay of 1.54 days/100 m for house wrens (*Troglodytes aedon*) in California. Altamirano et al. (2015) found the largest elevational effect, with a delay in fledging dates of 6.16 days/100 m for thorn‐tailed rayaditos (*Aphrastura spinicauda*) in an Andean temperate forest in Chile. Apart from this latter study, it therefore seems not unusual that there is only a slight delay in fledging dates at higher elevations.

However, elevational gradients in gonadal development may differ from those in fledging dates. In Canada, Bears (2007) found a delay of more than 30 days in breeding readiness (defined as the average date when the functional gonads had developed) of male dark‐eyed juncos (*Junco hyemalis*) at 2000 m compared to 1000 m. This delay in breeding readiness reversed when birds were kept under controlled environmental conditions (constant temperature and photoperiod, unlimited food supply). Birds captured at 2000 m and brought to the laboratory were 4–8 days earlier ready to breed compared to birds captured at 1000 m (Bears, 2007). Thus, dark‐eyed junco males captured at high elevation were ready to breed earlier compared to birds captured at low elevations when exposed to the same conditions (Bears, 2007) and males at high elevations initiated breeding at lower temperatures than their conspecifics at lower elevations. Our results may be explained by a similar mechanism as the one described by Bears (2007). If birds at high elevation initiated breeding at lower temperatures than their conspecifics at low elevations, the elevational gradient in the timing of the broods is steeper compared to the elevational gradient of the temperature threshold exceedance.

The bird species investigated in our study did not seem to adapt to the harsher environmental conditions at higher elevations by raising their brood later at a time when conditions were milder. Rather, such species may have evolved other adaptations to cope with the harsher conditions at higher elevations (Martin et al., 2023). For example, previous studies found morphological adaptations, where birds at higher elevations were smaller but had longer wings, tails or feathers (Bears, 1999, 2007; Lu et al., 2009; Sander & Chamberlain, 2020).

Further, birds living at higher elevations probably have evolved adaptations to cope with increased energy requirements, for example, increased parental investment. Badyaev & Ghalambor (2001) investigated the life history strategies of 24 pairs of bird species along an elevational gradient and found an increased male investment additionally to the female investment for species living at higher elevations compared to species living at lower elevations. Also, they found that birds at higher elevations raised fewer but higher‐quality offspring (Badyaev & Ghalambor, 2001). With increased parental investment at high elevations and fewer offspring, young birds may be able to grow faster and fledge earlier.

Another adaptation at higher elevations could be to build bigger or thicker nests: For example, Widmer (1993) found that Eurasian blackcaps (*Sylvia atricapilla*) built bigger and better insulated nests in the mountains compared to the lowlands. Blue tits also built bigger nests (Britt & Deeming, 2011) and changed the nest cup lining material (Mainwaring & Hartley, 2008) depending on temperatures during the nest‐building period. Britt and Deeming (2011) found clearer temperature‐related differences in nest size for blue tits than for great tits, and Schöll and Hille (2014) found no clear change in insulation of great tit nests along an elevational gradient (488–878 m). It therefore seems that temperature‐related differences in nest insulation or nest size are species‐specific.

Birds breeding at higher elevations are often not able to have a second brood due to a shortened season (Dillon & Conway, 2015; Labarbera & Lacey, 2018; Yeh & Price, 2004), which can make it difficult to compare breeding dates with lower elevations, where second broods may be possible. When birds have more than one brood, the observations of broods before and after fledging spread over a longer time, and therefore, it is more difficult to estimate the dates when 50% of the broods are certainly fledged; this is probably the main reason why for mistle thrushes and song thrushes, the estimated proportion of certainly fledged broods was lower than 100% at the end of the breeding season. Among our study species, especially song thrushes and mistle thrushes are known to often have more than one brood (Glutz von Blotzheim, 1988). For the mistle thrush, we could not obtain reasonable estimates for the mean date when broods were certainly fledged in 50% of observations, and mistle thrushes usually have more than one brood even at higher elevations. In contrast, song thrushes may have only one brood at medium and high elevations; a reason may be that the number of broods in song thrushes is more dependent on weather conditions (Glutz von Blotzheim, 1962; Mattes et al., 2005) than in mistle thrushes.

Eurasian chaffinches (Glutz von Blotzheim, 1997), coal tits (Glutz von Blotzheim & Bauer, 1993) and Alpine tits (Glutz von Blotzheim & Bauer, 1993) seem to usually have only one brood both at lower and higher elevations in the Alps. For coal tits and Alpine tits, we were able to estimate the mean date when broods became certainly fledged in 50% of observations at high, medium and low elevations. For the Eurasian chaffinch, it was more difficult to examine the mean date when broods were certainly fledged in 50% of observations at high elevation, because the number of observations was low. This may have been due to lower breeding density (Knaus et al., 2018) or lower breeding success of Eurasian chaffinches at higher elevations compared to coal tits and Alpine tits. Species with a main distribution in northern latitudes or high elevations such as coal tits and Alpine tits might be better adapted to harsher conditions than species with a main distribution in southern latitudes or lower elevations such as the Eurasian chaffinch.

Still, there may be differences in detection probabilities of feeding adults or begging young in different developmental stages that may have influenced the number of observations and thus our timing estimates. In our study, we did not take such detection probabilities into account, since to the best of our knowledge there are no published estimates on detection probabilities of broods of songbirds for different developmental stages of the young, which could be addressed in future studies. Our reported elevational gradients in the timing of the broods therefore rely on the assumption that differences in detection probability among the different breeding stages are not dependent on elevation.

In summary, and similar to previous studies, we found that songbirds delay breeding with increasing elevations to a lower degree than expected if they would breed at similar environmental conditions at all elevations. Therefore, the young of the investigated songbirds were raised under harsher environmental conditions at higher elevations and probably have evolved adaptations to cope with such conditions, rather than postponing breeding dates to warmer conditions. By using a dataset from a nationwide citizen science bird monitoring scheme, we were able to obtain estimates on the date when broods became certainly fledged without the need to search for nests and thus without causing disturbances at nests. In the future, it would be interesting to expand the study to a larger geographic region allowing for comparisons of mountain regions at different latitudes and of species with differing degrees of adaptions to living at high elevations and thus to increase our understanding of how bird species are able to adapt to living at higher elevations.

## AUTHOR CONTRIBUTIONS

**Julia Paterno:** Conceptualization (equal); data curation (equal); formal analysis (equal); funding acquisition (equal); methodology (equal); project administration (equal); validation (equal); visualization (equal); writing – original draft (equal); writing – review and editing (equal). **Fränzi Korner‐Nievergelt:** Conceptualization (equal); data curation (equal); formal analysis (equal); methodology (equal); project administration (equal); supervision (equal); validation (equal); visualization (equal); writing – original draft (equal); writing – review and editing (equal). **Stefanie Gubler:** Data curation (equal); formal analysis (equal); validation (equal); visualization (equal); writing – original draft (equal); writing – review and editing (equal). **Pia Anderwald:** Conceptualization (equal); funding acquisition (equal); methodology (equal); project administration (equal); supervision (equal); validation (equal); writing – original draft (equal); writing – review and editing (equal). **Valentin Amrhein:** Conceptualization (equal); data curation (equal); methodology (equal); project administration (equal); supervision (equal); validation (equal); visualization (equal); writing – original draft (equal); writing – review and editing (equal).

## CONFLICT OF INTEREST STATEMENT

The authors declare that there is no conflict of interest.

## Supporting information


Data S1.

Table S1.

Figure S1.

## Data Availability

Data and code can be found in Data S1.

## References

[ece370049-bib-0001] Altamirano, T. A. , Ibarra, J. T. , De La Maza, M. , Navarrete, S. A. , & Bonacic, C. (2015). Reproductive life‐history variation in a secondary cavity‐nester across an elevational gradient in Andean temperate ecosystems. Auk, 132(4), 826–835. 10.1642/AUK-15-28.1

[ece370049-bib-0002] Amrhein, V. , & Greenland, S. (2022). Discuss practical importance of results based on interval estimates and *p*‐value functions, not only on point estimates and null *p*‐values. Journal of Information Technology, 37(3), 316–320. 10.1177/02683962221105904

[ece370049-bib-0101] Badyaev, A. V. , & Ghalambor, C. K. (2001). Evolution of life histories along elevational gradients: Trade‐off between parental care and fecundity. Ecology, 82(10), 2948–2960. 10.1890/0012-9658(2001)082[2948:EOLHAE]2.0.CO;2

[ece370049-bib-0003] Bairlein, F. , Berthold, P. , Querner, U. , & Schlenker, R. (1980). Die Brutbiologie der Grasmücken *Sylvia atricapilla, borin, communis* und *curruca* in Mittel‐ und N‐Europa. Journal für Ornithologie, 121(4), 325–369. 10.1007/bf01643331

[ece370049-bib-0102] Bauer, H.‐G. , Bezzel, E. , & Fiedler, W. (2012). Das Kompendium der Vögel Mitteleuropas (2nd ed.). AULA‐Verlag Wiesbaden.

[ece370049-bib-0004] BDM Coordination Office . (2014). Swiss biodiversity monitoring BDM. Description of methods and indicators. Environmental Studies, 1410, 103.

[ece370049-bib-0005] Bears, H. (2007). Elevation and the avian phenotype: field and experimental studies of breeding dark‐eyed juncos. *PhD Thesis* .

[ece370049-bib-0007] Betancourt, M. (2013). Generalizing the No‐U‐Turn sampler to Riemannian manifolds. *arXiv 1304*, Nr. 1920.

[ece370049-bib-0103] Blanco, G. , Fargallo, J. A. , Cuevas, J. A. , & Tella, J. L. (1998). Effects of nest‐site availability and distribution on density‐dependent clutch size and laying date in the Chough *Pyrrhocorax pyrrhocorax* . Ibis, 140(2), 252–256. 10.1111/j.1474-919x.1998.tb04386.x

[ece370049-bib-0009] Brändli, U. B. (1998). Die haufigsten Waldbaume der Schweiz. Ergebnisse aus dem Landesforstinventar 1983–85: Verbreitung, Standort und Haufigkeit von 30 Baumarten. In Berichte – Eidgenossischen Forschungsanstalt fur Wald, Schnee und Landschaft, 342. Eidgenössische Foschungsanstalt für Wald, Schnee und Landschaft, Birmensdorf.

[ece370049-bib-0010] Britt, J. , & Deeming, D. C. (2011). First‐egg date and air temperature affect nest construction in blue tits *Cyanistes caeruleus*, but not in great tits *Parus major* . Bird Study, 58(1), 78–89. 10.1080/00063657.2010.524916

[ece370049-bib-0011] Brügger, R. , & Vasella, A. (2003). Pflanzen im Wandel der Jahreszeiten, Anleitung für phänologische Beobachtungen/Les plantes au cours des saisons. Guide pour observation phénologiques (p. 288). Geographica Bernensia.

[ece370049-bib-0012] Carpenter, B. , Gelman, A. , Hoffman, M. D. , Lee, D. , Goodrich, B. , Betancourt, M. , Brubaker, M. , Guo, J. , Li, P. , & Riddell, A. (2017). Stan: A probabilistic programming language. Journal of Statistical Software, 76(1), 1–32. 10.18637/jss.v076.i01 36568334 PMC9788645

[ece370049-bib-0104] Clouet, M. (2005). Breeding biology of Coal Tit *Parus ater* in Central Pyrenees. Alauda, 73(2), 81–90.

[ece370049-bib-0014] Daan, S. , Dijkstra, C. , Drent, R. , & Meijer, T. (1989). Food supply and the annual timing of avian reproduction. *Acta XIX Congressus Internationalis Ornithologici, Volume I. 19th International Ornithological Congress*, *I*, 392–407.

[ece370049-bib-0105] Delestrade, A. (2002). Breeding biology and distribution of the Northern Raven *Corvus corax* in Corsica. Alauda revue internationale d'ornithologie: Revue trimestrielle de la Société d'Etudes Ornithologiques de France, 70(2), 293–300.

[ece370049-bib-0015] Dillon, K. G. , & Conway, C. J. (2015). Elevational gradient in clutch size of red‐faced warblers. Journal of Field Ornithology, 86(2), 163–172. 10.1111/jofo.12099

[ece370049-bib-0106] Driver, J. (2005). A study of breeding carrion crows *Corvus corone* in Snowdonia. Welsh Birds, 4(3), 227–235.

[ece370049-bib-0016] Dunn, P. O. , Thusius, K. J. , Kimber, K. , & Winkler, D. W. (2000). Geographic and ecological variation in clutch size of tree swallows. Auk, 117(1), 215–221. 10.1642/0004-8038(2000)117[0215:GAEVIC]2.0.CO;2

[ece370049-bib-0017] Fontanilles, P. (2022). Abundance, reproduction and habitat of the whinchat *Saxicola rubetra* in the Pyrenees: Compatibility with mowing and grazing practices. Alauda, 90(3), 215–232.

[ece370049-bib-0019] Frei, C. (2013). Interpolation of temperature in a mountainous region using nonlinear profiles and non‐Euclidean distances. International Journal of Climatology, 34(5), 1585–1605. 10.1002/joc.3786

[ece370049-bib-0021] Gibbs, H. (2007). Climatic variation and breeding in the Australian Magpie (*Gymnorhina tibicen*): A case study using existing data. Emu, 107(4), 284–293. 10.1071/MU07022

[ece370049-bib-0022] Gil‐Delgado, J. A. , Lopez, G. , & Barba, E. (1992). Breeding ecology of the blue tit *Parus caeruleus* in eastern Spain: A comparison with other localities with special reference to Corsica. Ornis Scandinavica, 23(4), 444–450. 10.2307/3676675

[ece370049-bib-0023] Glutz von Blotzheim, U. (1962). Die Brutvögel der Schweiz. Verlag Aargauer Tagblatt.

[ece370049-bib-0024] Glutz von Blotzheim, U. (1988). II Passeriformes (2. Teil) Turdidae. In Handbuch der Vögel Mitteleuropas (Vol. 11, p. 1226). Akademische Verlagsgesellschaft.

[ece370049-bib-0025] Glutz von Blotzheim, U. (1997). II: Passeriformes (5.Teil) Fringillidae (Vol 14). In Handbuch der Vögel Mitteleuropas (Vol. 14, p. 1242). Akademische Verlagsgesellschaft.

[ece370049-bib-0026] Glutz von Blotzheim, U. , & Bauer, K. M. (1993). I: Passeriformes (4. Teil) Muscicapidae – Paridae. In Handbuch der Vögel Mitteleuropas (Vol. 13, p. 808). Akademische Verlagsgesellschaft.

[ece370049-bib-0027] Hinks, A. E. , Cole, E. F. , Daniels, K. J. , Wilkin, T. A. , Nakagawa, S. , & Sheldon, B. C. (2015). Scale‐dependent phenological synchrony between songbirds and their Caterpillar food Aource. American Naturalist, 186(1), 84–97. 10.1086/681572 26098341

[ece370049-bib-0028] Illán, J. G. , Gutiérrez, D. , Díez, S. B. , & Wilson, R. J. (2012). Elevational trends in butterfly phenology: Implications for species responses to climate change. Ecological Entomology, 37(2), 134–144. 10.1111/j.1365-2311.2012.01345.x

[ece370049-bib-0030] Johnson, L. S. , Iser, K. M. , Molnar, H. A. , Nguyen, A. V. , & Connor, C. L. (2018). Clutch and egg size of tree swallows along an elevational gradient. Journal of Field Ornithology, 89(3), 234–241. 10.1111/jofo.12262

[ece370049-bib-0108] Johnson, L. S. , Ostlind, E. , Brubaker, J. L. , Balenger, S. L. , Johnson, B. G. P. , & Golden, H. (2006). Changes in egg size and clutch size with elevation in a Wyoming population of Mountain Bluebirds. Condor, 108(3), 591–600. 10.1650/0010-5422(2006)108[591:CIESAC]2.0.CO;2

[ece370049-bib-0031] Keller, V. , Herrando, S. , Voříšek, P. , Franch, M. , Kipson, M. , Milanesi, P. , Martí, D. , Anton, M. , Klvaňová, A. , Kalyakin, M. V. , Bauer, H.‐G. , & Foppen, R. P. B. (2020). European breeding bird atlas 2: Distribution, Abundance and Change. European Bird Census Council & Lynx Edicions.

[ece370049-bib-0109] Kleindorfer, S. (2007). The ecology of clutch size variation in Darwin's Small Ground Finch *Geospiza fuliginosa*: Comparison between lowland and highland habitats. Ibis, 149(4), 730–741. 10.1111/j.1474-919X.2007.00694.x

[ece370049-bib-0032] Knaus, P. , Antoniazza, S. , Wechsler, S. , Guélat, J. , Kery, M. , Strebel, N. , & Sattler, T. (2018). Schweizer Brutvogelatlas 2013–2016. Verbreitung und Bestandsentwicklung der Vögel in der Schweiz und im Fürstentum Liechtenstein. Schweizerische Vogelwarte Sempach.

[ece370049-bib-0033] Korner‐Nievergelt, F. , & Robinson, R. (2019). Birdring: Methods to analyse ring re‐encounter data. R Package Version 1.4 . https://CRAN.R‐project.org/package=birdring

[ece370049-bib-0035] Labarbera, K. , & Lacey, E. A. (2018). Breeding season length and nest mortality drive cryptic life history variation in dark‐eyed juncos (*Junco hyemalis*) breeding across a montane elevational gradient. Auk, 135(2), 284–298. 10.1642/AUK-17-184.1

[ece370049-bib-0036] Lack, D. (1950). The breeding seasons of European birds. Ibis, 92, 288–316.

[ece370049-bib-0038] Levin, R. N. , Correa, S. M. , Freund, K. A. , & Fuxjager, M. J. (2023). Latitudinal and elevational variation in the reproductive biology of house wrens. Troglodytes Aedon. Ecology and Evolution, 13(9), 1–15. 10.1002/ece3.10476 PMC10495810 37706165

[ece370049-bib-0110] Logie, J. W. (1998). Population ecology and lifetime reproductive success of dippers Cinclus cinclus . [Doctoral dissertation]. Universtiy of Stirling.

[ece370049-bib-0111] Lu, X. , Ke, D. H. , Zeng, X. H. , & Yu, T. L. (2009). Reproductive ecology of two sympatric Tibetan snowfinch species at the edge of their altitudinal range: Response to more stressful environments. Journal of Arid Environments, 73(12), 1103–1108. 10.1016/j.jaridenv.2009.06.011

[ece370049-bib-0039] Lu, X. , Yu, T. , Liang, W. , & Yang, C. (2010). Comparative breeding ecology of two White‐bellied redstart populations at different altitudes. Journal of Field Ornithology, 81(2), 167–175. 10.1111/j.1557-9263.2010.00274.x

[ece370049-bib-0040] Mainwaring, M. C. , & Hartley, I. R. (2008). Seasonal adjustments in nest cup lining in blue tits *Cyanistes caeruleus* . Ardea, 96(2), 278–282. 10.5253/078.096.0213

[ece370049-bib-0041] Martin, K. , de Zwaan, D. R. , Scridel, D. , & Altamirano, T. A. (2023). 2. Avian adaptations to High Mountain habitats. In D. E. Chamberlain , A. Lehikoinen , & K. Martin (Eds.), Ecology and conservation of mountain birds (pp. 35–89). Cambridge University Press.

[ece370049-bib-0042] Mattes, H. , Maurizio, R. , & Bürkli, W. (2005). Die Vogelwet im Oberengadin, Bergell und Puschlav. Ein Naturführer zur Avifauna in einem inneralpinen Gebiet. Schweizerische Vogelwarte Sempach.

[ece370049-bib-0044] Müller, M. , Spaar, R. , Schifferli, L. , & Jenni, L. (2005). Effects of changes in farming of subalpine meadows on a grassland bird, the whinchat (*Saxicola rubetra*). Journal of Ornithology, 146(1), 14–23. 10.1007/s10336-004-0059-0

[ece370049-bib-0113] Ogden, L. J. E. , Martin, M. , & Martin, K. (2012). Mating and breeding success decline with elevation for the pacific wren (*Troglodytes pacificus*) in coastal mountain forests. Wilson Journal Of Ornithology, 124(2), 270–276. 10.1676/11-186.1

[ece370049-bib-0114] Ormerod, S. J. , & Tyler, S. J. (1987). Aspects of the breeding ecology of welsh grey wagtails *Motacilla cinerea* . Bird Study, 34(1), 43–51. 10.1080/00063658709476935

[ece370049-bib-0045] Ornitho (2023). www.ornitho.ch

[ece370049-bib-0047] Pereyra, M. E. (2011). Effects of snow‐related environmental variation on breeding schedules and productivity of a high‐altitude population of dusky flycatchers (*Empidonax oberholseri*). The Auk, 128(4), 746–758.

[ece370049-bib-0048] Perfito, N. , Tramontin, A. D. , Meddle, S. , Sharp, P. , Afik, D. , Gee, J. , Ishii, S. , Kikuchi, M. , & Wingfield, J. C. (2004). Reproductive development according to elevation in a seasonally breeding male songbird. Oecologia, 140(2), 201–210. 10.1007/s00442-004-1576-5 15148599

[ece370049-bib-0050] R Core Team . (2020). R: A language and environment for statistical computing. R Foundation for Statistical Computing. https://www.r‐project.org/

[ece370049-bib-0115] Sander, M. M. , & Chamberlain, D. (2020). Evidence for intraspecific phenotypic variation in songbirds along elevation gradients in central Europe. Ibis, 162(4), 1355–1362. 10.1111/ibi.12843

[ece370049-bib-0051] Saracco, J. F. , Siegel, R. B. , Helton, L. , Stock, S. L. , & DeSante, D. F. (2018). Phenology and productivity in a montane bird assemblage: Trends and responses to elevation and climate variation. Global Change Biology, 25(3), 985–996. 10.1111/gcb.14538 30506620

[ece370049-bib-0052] Schano, C. , Niffenegger, C. , Jonas, T. , & Korner‐Nievergelt, F. (2021). Hatching phenology is lagging behind an advancing snowmelt pattern in a high‐alpine bird. Scientific Reports, 11, 22191. 10.1038/s41598-021-01497-8 34772973 PMC8589975

[ece370049-bib-0053] Schöll, E. M. , & Hille, S. M. (2014). Do great tits *Parus major* nesting at high altitudes build better insulated nests? Ardeola, 61(2), 323–333. 10.13157/arla.61.2.2014.323

[ece370049-bib-0116] Schweizerische Vogelwarte Sempach . (2019). Avifaunistik‐Merkblatt. https://www.vogelwarte.ch/modx/assets/files/projekte/ueberwachung/id/Aussergewoehnliche_Beobachtungen_d.pdf

[ece370049-bib-0054] Smith, K. G. , & Andersen, D. C. (2014). Snowpack and variation in reproductive ecology of a montane ground‐nesting passerine, *Junco hyemalis* . Ornis Scandinavica, 16(1), 8–13.

[ece370049-bib-0055] Stan Development Team . (2023). RStan: The R interface to Stan. R package version 2.32.3 . https://mc‐stan.org/

[ece370049-bib-0056] Stier, A. , Delestrade, A. , Zahn, S. , Arrivé, M. , Criscuolo, F. , & Massemin‐Challet, S. (2014). Elevation impacts the balance between growth and oxidative stress in coal tits. Oecologia, 175(3), 791–800. 10.1007/s00442-014-2946-2 24805201

[ece370049-bib-0057] Swiss Ornithological Institute . (2019). Avifaunistik‐Merkblatt. Swiss Ornithological Institute.

[ece370049-bib-0117] Tavecchia, G. , Pradel, R. , Lebreton, J.‐D. , Biddau, L. , & Mingozzi, T. (2002). Sex‐biased survival and breeding dispersal probability in a patchy population of the Rock Sparrow *Petronia petronia* . Ibis, 144(2), E79–E87. 10.1046/j.1474-919x.2002.00059.x

[ece370049-bib-0058] Thackeray, S. J. , Henrys, P. A. , Hemming, D. , Bell, J. R. , Botham, M. S. , Burthe, S. , Helaouet, P. , Johns, D. G. , Jones, I. D. , Leech, D. I. , MacKay, E. B. , Massimino, D. , Atkinson, S. , Bacon, P. J. , Brereton, T. M. , Carvalho, L. , Clutton‐Brock, T. H. , Duck, C. , Edwards, M. , … Wanless, S. (2016). Phenological sensitivity to climate across taxa and trophic levels. Nature, 535(7611), 241–245. 10.1038/nature18608 27362222

[ece370049-bib-0059] Verhagen, I. , Tomotani, B. M. , Gienapp, P. , & Visser, M. E. (2020). Temperature has a causal and plastic effect on timing of breeding in a small songbird. Journal of Experimental Biology, 223(8), 1–7. 10.1242/jeb.218784 32205357

[ece370049-bib-0060] Visser, M. E. , & Both, C. (2005). Shifts in phenology due to global climate change: The need for a yardstick. Proceedings of the Royal Society B: Biological Sciences, 272(1581), 2561–2569. 10.1098/rspb.2005.3356 PMC1559974 16321776

[ece370049-bib-0061] Visser, M. E. , & Holleman, L. J. M. (2001). Warmer springs disrupt the synchrony of oak and winter moth phenology. Proceedings of the Royal Society B: Biological Sciences, 268(1464), 289–294. 10.1098/rspb.2000.1363 PMC1088605 11217900

[ece370049-bib-0062] Vitasse, Y. , Ursenbacher, S. , Klein, G. , Bohnenstengel, T. , Chittaro, Y. , Delestrade, A. , Monnerat, C. , Rebetez, M. , Rixen, C. , Strebel, N. , Schmidt, B. R. , Wipf, S. , Wohlgemuth, T. , Yoccoz, N. G. , & Lenoir, J. (2021). Phenological and elevational shifts of plants, animals and fungi under climate change in the European Alps. Biological Reviews, 96(5), 1816–1835. 10.1111/brv.12727 33908168

[ece370049-bib-0063] Widmer, M. (1993). Brutbiologie einer Gebirgspopulation der Gartengrasmücke *Sylvia borin* . Ornithologische Beobachter, 90(2), 85–113.

[ece370049-bib-0064] Williams, T. D. (2012). Physiological adaptations for breeding in birds. Princeton University Press.

[ece370049-bib-0065] Yeh, P. J. , & Price, T. D. (2004). Adaptive phenotypic plasticity and the successful colonization of a novel environment. American Naturalist, 164(4), 531–542. 10.1086/423825 15459883

[ece370049-bib-0107] Zang, H. (1980). Der Einfluß der Höhenlage auf Siedlungsdichte und Brutbiologie höhlenbrütender Singvögel im Harz. Journal of Ornithology, 121(4), 371–386. 10.1007/bf01643332

[ece370049-bib-0207] Zang, H. (1982). Der Einfluß der Höhenlage auf Alterszusammensetzung und Brutbiologie bei Kohl‐ und Blaumeise (*Parus major*, *P. caeruleus*) im Harz. Journal of Ornithology, 123, 145–154. 10.1007/BF01645054

