# Supplementary material for: Alpine songbirds at higher elevations are only raised with a slight delay and therefore under harsher environmental conditions
Source: Ecol Evol. 2024 Jul 25;14(7):e70049. doi: 10.1002/ece3.70049 (PMC11272606; doi:10.1002/ece3.70049)
Supplement: Supplementary file 2 — Table S1. [file ECE3-14-e70049-s003.docx]

Table S1 Number of observations per species, year and elevational belt.

| species | year | observations ‘certainly or likely not fledged’ | | | | Observations ‘certainly fledged’ | | | | observations ‘certainly or likely not fledged’ and ‘certainly fledged’ |
| --- | --- | --- | --- | --- | --- | --- | --- | --- | --- | --- |
|  |  | 1500-1733 | 1734-1967 | 1968-2200 | total | 1500-1733 | 1734-1967 | 1968-2200 | total |  |
| coal tit | 2013 | 15 | 7 | 4 | **26** | 6 | 4 | 2 | **12** | 38 |
|  | 2014 | 28 | 17 | 3 | **48** | 23 | 35 | 8 | **66** | 114 |
|  | 2015 | 30 | 16 | 7 | **53** | 46 | 33 | 15 | **94** | 147 |
|  | 2016 | 17 | 10 | 5 | **32** | 31 | 25 | 9 | **65** | 97 |
|  | 2017 | 12 | 9 | 3 | **24** | 15 | 8 | 5 | **28** | 52 |
|  | 2018 | 8 | 5 | 3 | **16** | 17 | 14 | 4 | **35** | 51 |
|  | 2019 | 8 | 6 | 3 | **17** | 18 | 18 | 4 | **40** | 57 |
|  | 2020 | 13 | 10 | 2 | **25** | 9 | 14 | 4 | **27** | 52 |
|  | 2021 | 19 | 9 | 3 | **31** | 26 | 13 | 3 | **42** | 73 |
|  | 2022 | 11 | 6 | 1 | **18** | 31 | 14 | 2 | **47** | 65 |
|  | total | **161** | **95** | **34** | **290** | **222** | **178** | **56** | **456** | **746** |
| Alpine/ willow tit | 2013 | 5 | 5 | 4 | **14** | 5 | 9 | 2 | **16** | 30 |
|  | 2014 | 6 | 15 | 4 | **25** | 11 | 10 | 8 | **29** | 54 |
|  | 2015 | 7 | 4 | 2 | **13** | 12 | 11 | 8 | **31** | 44 |
|  | 2016 | 5 | 2 | 3 | **10** | 4 | 8 | 8 | **20** | 30 |
|  | 2017 | 2 | 3 | 2 | **7** | 2 | 2 | 0 | **4** | 11 |
|  | 2018 | 2 | 1 | 4 | **7** | 1 | 7 | 5 | **13** | 20 |
|  | 2019 | 3 | 4 | 0 | **7** | 2 | 3 | 0 | **5** | 12 |
|  | 2020 | 3 | 3 | 3 | **9** | 2 | 5 | 2 | **9** | 18 |
|  | 2021 | 4 | 4 | 0 | **8** | 1 | 4 | 0 | **5** | 13 |
|  | 2022 | 5 | 4 | 2 | **11** | 7 | 7 | 1 | **15** | 26 |
|  | total | **42** | **45** | **24** | **111** | **47** | **66** | **34** | **147** | **258** |
| Eurasian chaffinch | 2013 | 0 | 5 | 0 | **5** | 2 | 3 | 1 | **6** | 11 |
|  | 2014 | 6 | 8 | 1 | **15** | 2 | 6 | 0 | **8** | 23 |
|  | 2015 | 7 | 11 | 4 | **22** | 7 | 5 | 2 | **14** | 36 |
|  | 2016 | 16 | 9 | 7 | **32** | 9 | 8 | 5 | **22** | 54 |
|  | 2017 | 2 | 2 | 2 | **6** | 1 | 4 | 1 | **6** | 12 |
|  | 2018 | 2 | 3 | 0 | **5** | 9 | 8 | 2 | **19** | 24 |
|  | 2019 | 4 | 4 | 0 | **8** | 6 | 5 | 3 | **14** | 22 |
|  | 2020 | 6 | 5 | 1 | **12** | 7 | 8 | 1 | **16** | 28 |
|  | 2021 | 10 | 5 | 1 | **16** | 9 | 3 | 2 | **14** | 30 |
|  | 2022 | 10 | 2 | 0 | **12** | 11 | 1 | 0 | **12** | 24 |
|  | total | **63** | **54** | **16** | **133** | **63** | **51** | **17** | **131** | **264** |
| song thrush | 2013 | 5 | 2 | 0 | **7** | 0 | 4 | 0 | **4** | 11 |
|  | 2014 | 4 | 1 | 0 | **5** | 5 | 5 | 1 | **11** | 16 |
|  | 2015 | 5 | 3 | 0 | **8** | 9 | 3 | 1 | **13** | 21 |
|  | 2016 | 28 | 5 | 4 | **37** | 13 | 5 | 4 | **22** | 59 |
|  | 2017 | 2 | 1 | 0 | **3** | 1 | 1 | 0 | **2** | 5 |
|  | 2018 | 5 | 0 | 1 | **6** | 5 | 2 | 1 | **8** | 14 |
|  | 2019 | 2 | 7 | 1 | **10** | 3 | 4 | 3 | **10** | 20 |
|  | 2020 | 4 | 2 | 3 | **9** | 7 | 4 | 1 | **12** | 21 |
|  | 2021 | 2 | 2 | 1 | **5** | 3 | 0 | 0 | **3** | 8 |
|  | 2022 | 4 | 4 | 1 | **9** | 6 | 2 | 0 | **8** | 17 |
|  | total | **61** | **27** | **11** | **99** | **52** | **30** | **11** | **93** | **192** |
| mistle thrush | 2013 | 17 | 20 | 2 | **39** | 4 | 7 | 0 | **11** | 50 |
|  | 2014 | 8 | 12 | 6 | **26** | 7 | 7 | 8 | **22** | 48 |
|  | 2015 | 17 | 12 | 5 | **34** | 11 | 9 | 5 | **25** | 59 |
|  | 2016 | 36 | 17 | 13 | **66** | 15 | 6 | 4 | **25** | 91 |
|  | 2017 | 3 | 13 | 1 | **17** | 5 | 5 | 6 | **16** | 33 |
|  | 2018 | 9 | 7 | 4 | **20** | 10 | 5 | 0 | **15** | 35 |
|  | 2019 | 11 | 9 | 10 | **30** | 8 | 4 | 3 | **15** | 45 |
|  | 2020 | 15 | 15 | 2 | **32** | 17 | 6 | 3 | **26** | 58 |
|  | 2021 | 16 | 8 | 4 | **28** | 5 | 10 | 0 | **15** | 43 |
|  | 2022 | 19 | 10 | 6 | **35** | 7 | 11 | 5 | **23** | 58 |
|  | total | **151** | **123** | **53** | **327** | **89** | **70** | **34** | **193** | **520** |
