# Supplementary material for: Alpine songbirds at higher elevations are only raised with a slight delay and therefore under harsher environmental conditions
Source: Ecol Evol. 2024 Jul 25;14(7):e70049. doi: 10.1002/ece3.70049 (PMC11272606; doi:10.1002/ece3.70049)
Supplement: Supplementary file 3 — Figure S1. [file ECE3-14-e70049-s002.docx]

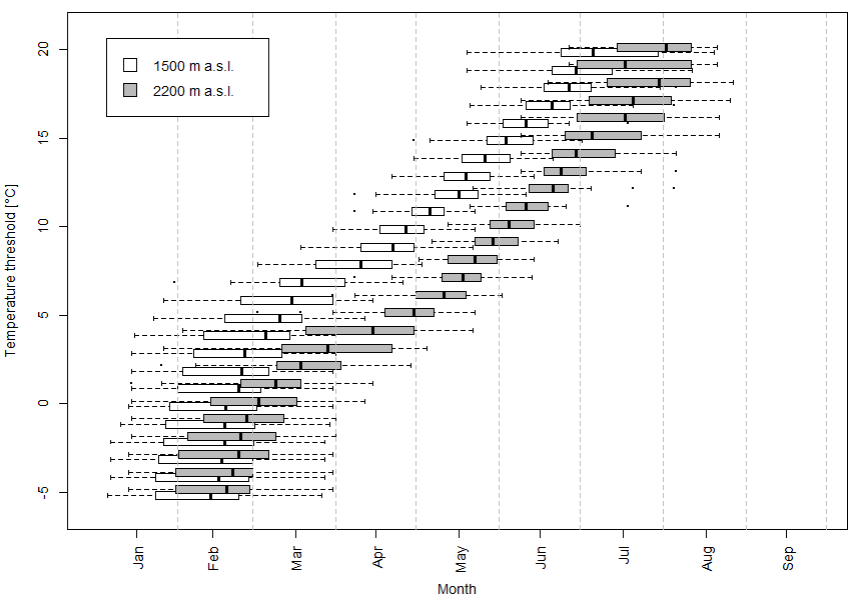


Figure S1 Differences in dates when a certain temperature threshold was reached at 2200 compared to 1500 m.
